# Immunogenicity of a first dose of mRNA- or vector-based SARS-CoV-2 vaccination in dialysis patients: a multicenter prospective observational pilot study

**DOI:** 10.1007/s40620-021-01076-0

**Published:** 2021-05-29

**Authors:** Paul Lesny, Mark Anderson, Gavin Cloherty, Michael Stec, Anja Haase-Fielitz, Mathias Haarhaus, Carla Santos, Carlos Lucas, Fernando Macario, Michael Haase

**Affiliations:** 1 Diaverum Renal Care Center, 14469 Potsdam, Germany; 2 Abbott Infectious Disease Research, Chicago, IL 60064-3500 USA; 3 Brandenburg Medical School Theodor Fontane, 16816 Neuruppin, Germany; 4 Faculty of Health Sciences Brandenburg, 14469 Potsdam, Germany; 5 Institute of Integrated Health Care Systems Research & Social Medicine, Otto-von-Guericke-University Magdeburg, 39120 Magdeburg, Germany; 6 Department of Cardiology, Brandenburg Heart Center, Immanuel Hospital, 16321 Bernau, Germany; 7 Diaverum AB, 21532 Malmö, Sweden; 8 Division of Renal Medicine, Department of Clinical Sciences, Intervention and Technology, Karolinska Institutet, Karolinska University Hospital, 17177 Stockholm, Sweden; 9 Cardiovascular Research and Development Unit, Faculty of Medicine, 4200-319 Porto, Portugal; 10 Medical Faculty, Otto-von-Guericke University Magdeburg, Leipziger Str. 44, 39120 Magdeburg, Germany

**Keywords:** mRNA- or vector-based SARS-CoV-2 vaccination, Responder, Hemodialysis, Peritoneal dialysis, COVID-19, ACE2 receptor binding inhibition capacity

## Abstract

**Background:**

Dialysis patients are at risk for lower SARS-CoV-2-vaccine immunogenicity than the normal population. We assessed immunogenicity to a first mRNA- or vector-based SARS-CoV-2-vaccination dose in dialysis patients.

**Methods:**

In a multicenter observational pilot study, 2 weeks after a first vaccination (BNT162b2/Pfizer-BioNTech [Comirnaty] or ChAdOx1 nCoV-19/Oxford-Astra-Zeneca [Vaxzevria]), hemodialysis patients (N = 23), peritoneal dialysis patients (N = 4) and healthy staff (N = 14) were tested for SARS-CoV-2-spike IgG/IgM, Nucleocapsid-protein-IgG-antibodies and plasma ACE2-receptor-binding-inhibition capacity. Hemodialysis patients who had had prior COVID-19 infection (N = 18) served as controls. Both response to first SARS-CoV-2 vaccination and IgG spike-positivity following prior COVID-19 infection were defined as SARS-CoV-2 spike IgG levels ≥ 50 AU/mL.

**Results:**

Vaccination responder rates were 17.4% (4/23) in hemodialysis patients, 100% (4/4) in peritoneal dialysis patients and 57.1% (8/14) in staff (HD vs. PD: p = 0.004, HD vs. staff: p = 0.027). Among hemodialysis patients, type of vaccine (Comirnaty N = 11, Vaxzevria N = 12, 2 responders each) did not appear to influence antibody levels (IgG spike: Comirnaty median 0.0 [1.–3. quartile 0.0–3.8] versus Vaxzevria 4.3 [1.6–20.1] AU/mL, p = 0.079). Of responders to the first dose of SARS-CoV-2 vaccination among hemodialysis patients (N = 4/23), median IgG spike levels and ACE2-receptor-binding-inhibition capacity were lower than that of IgG spike-positive hemodialysis patients with prior COVID-19 infection (13/18, 72.2%): IgG spike: median 222.0, 1.–3. quartile 104.1–721.9 versus median 3794.6, 1.–3. quartile 793.4–9357.9 AU/mL, p = 0.015; ACE2-receptor-binding-inhibition capacity: median 11.5%, 1.–3. quartile 5.0–27.3 versus median 74.8%, 1.–3. quartile 44.9–98.1, p = 0.002.

**Conclusions:**

Two weeks after their first mRNA- or vector-based SARS-CoV-2 vaccination, hemodialysis patients demonstrated lower antibody-related response than peritoneal dialysis patients and healthy staff or unvaccinated hemodialysis patients following prior COVID-19 infection.

**Graphic abstract:**

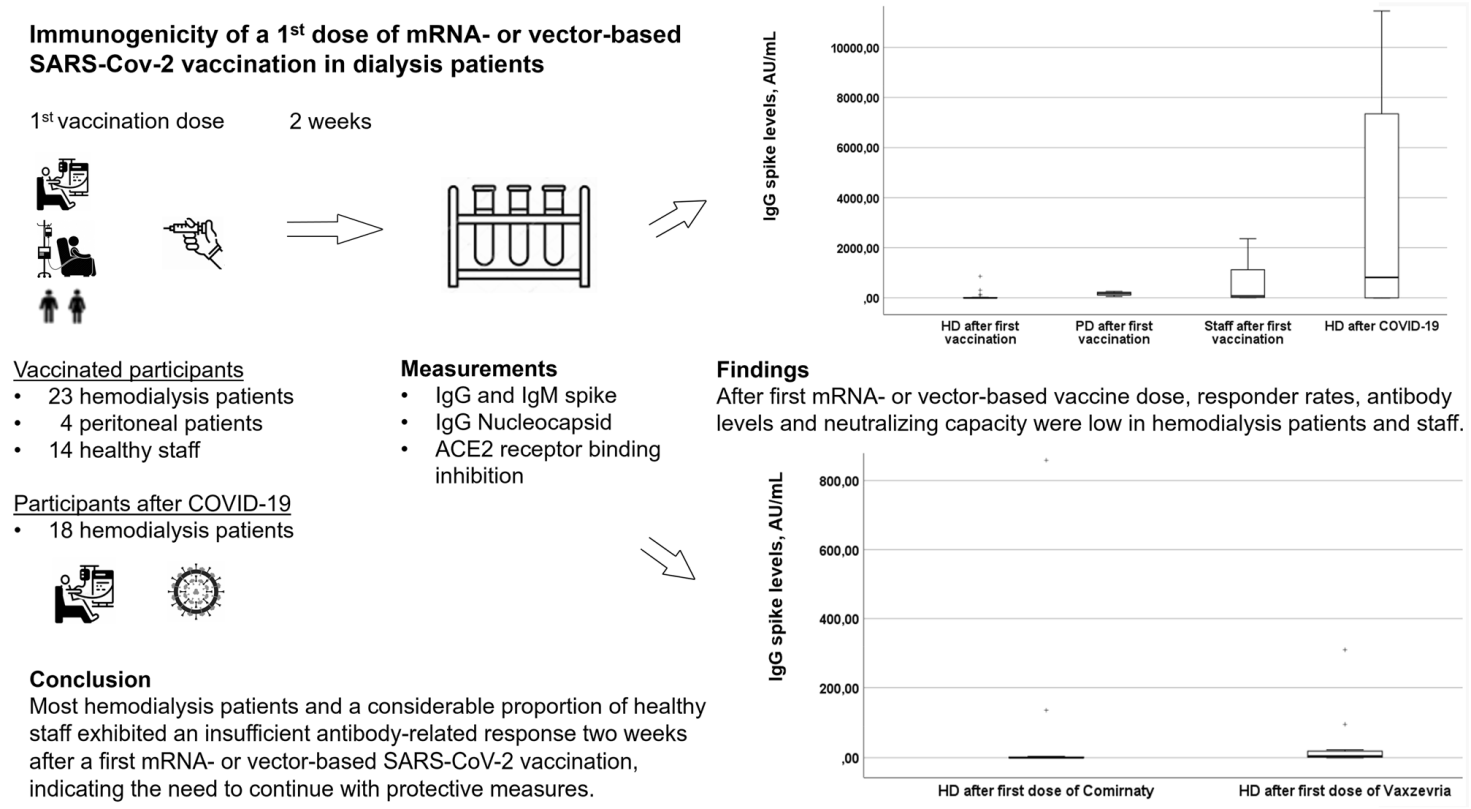

## Introduction

Several two-dose SARS-CoV-2 vaccines have been approved to prevent COVID-19 infection, with a reported vaccine efficacy of 90–95% in the normal population after the second dose. Dialysis patients are at high risk for COVID-19 infection and mortality [1, 2] but were not included in the vaccination registration trials. Small observational studies showed a sufficient immune response several weeks after the second SARS-CoV-2 mRNA-based vaccination [3, 4]. However, no study compared the effect of a first mRNA-based vaccination with that of a vector-based vaccination on antibody response in hemodialysis patients.

In a pilot study, we aimed to assess the antibody-related immunogenicity of a first dose of mRNA- or vector-based SARS-CoV-2 vaccine in this patient population compared to that of peritoneal dialysis patients, staff and unvaccinated hemodialysis patients who had had a prior COVID-19 infection.

## Methods

### Design, setting and participants

In a multicenter, prospective, observational pilot study, we tested blood antibody levels to the SARS-CoV-2 Spike (S-protein) and nucleocapsid (N-protein) proteins in hemodialysis, peritoneal dialysis, or healthy control populations (health care workers/staff) at the Diaverum Renal Care Centers Potsdam, Ludwigsfelde and Rangsdorf, receiving the BNT162b2/Pfizer-BioNTech (Comirnaty) or ChAdOx1 nCoV-19/Oxford-Astra-Zeneca (Vaxzevria) vaccine, and in hemodialysis patients having previously presented with PCR-positive COVID-19. Approval was obtained from the Ethics Committee of ‘*Landesärztekammer Brandenburg’*, Germany (registry number S9/(bB)/2021). The procedures used in this study adhere to the tenets of the Declaration of Helsinki. Written informed consent to participate and publish was obtained from all individual study participants. Information regarding clinical data was collected from medical records. Healthy controls provided demographic data. This manuscript adheres to the *‘Strengthening the Reporting of Observational Studies in Epidemiology’* guidelines [5].

### SARS-CoV-2 antibodies and ACE2-receptor-binding-inhibition capacity

Participant plasma was collected at baseline and 2 weeks after receiving a first vaccine dose. Participants were tested for SARS-CoV-2 IgG and IgM antibodies directed against the S-protein, and IgG antibodies directed against the SARS-CoV-2N-protein. All samples were run on Abbott ARCHITECT™ *i*2000SR instrument (Abbott Park, IL). The FDA EUA approved SARS-CoV-2 IgG (List 6R86), AdviseDx SARS-CoV-2 IgM (List 6R87), and SARS-CoV-2 IgG II Quant (List 6S60) assays were used, both automated Chemiluminescent Microparticle Immunoassays (CMIA). Assay results are reported as an index value of the ratio of specimen to calibrator Relative Light Units (RLU) signal. The SARS-CoV-2 IgG II Quant assay is an automated CMIA used for quantitative detection of IgG antibodies directed against the receptor-binding-domain of the SARS-CoV-2 S-protein. Assay linearity was shown between 21.0 and 40,000 AU/mL. A Research Use Only automated CMIA assay measured the capacity of SARS-CoV-2 antibodies, present in participant plasma, to inhibit SARS-CoV-2 receptor-binding-domain from binding to ACE2-receptors. The laboratory investigators were blinded to the sample sources and clinical outcomes. Researchers who obtained clinical data were blinded to antibody measurements.

### Study endpoints

SARS-CoV-2 spike IgG, IgM and Nucleocapsid IgG levels (AU/mL) and ACE2-receptor-binding-inhibition capacity (%) were provided as linear variables. Both response to the first SARS-CoV-2 vaccination and antibody level positivity following prior COVID-19 infection were defined as SARS-CoV-2 spike IgG levels ≥ 50 AU/mL.

### Statistical analysis

Study size was determined by the first badge of antibody measurements to gather early potentially important clinical information for this patient population. Values are presented as median (1.–3. quartile). Antibody levels and response status were compared: (i) hemodialysis patients receiving the mRNA-based SARS-CoV-2 vaccination vs. hemodialysis patients receiving the vector-based vaccine, (ii) vaccinated hemodialysis patients versus hemodialysis patients following prior COVID-19 infection, (iii) vaccinated hemodialysis versus peritoneal patients and (iv) vaccinated hemodialysis patients versus staff. Mann–Whitney-*U-*test, χ^2^ test, or Fisher’s exact test were used where appropriate. Alpha was set at 0.05 (2-tailed). SPSS, version 26.0 (IBM Corp., Armonk, NY, USA) was used.

## Results

### Participant characteristics

**Fig. 1:**
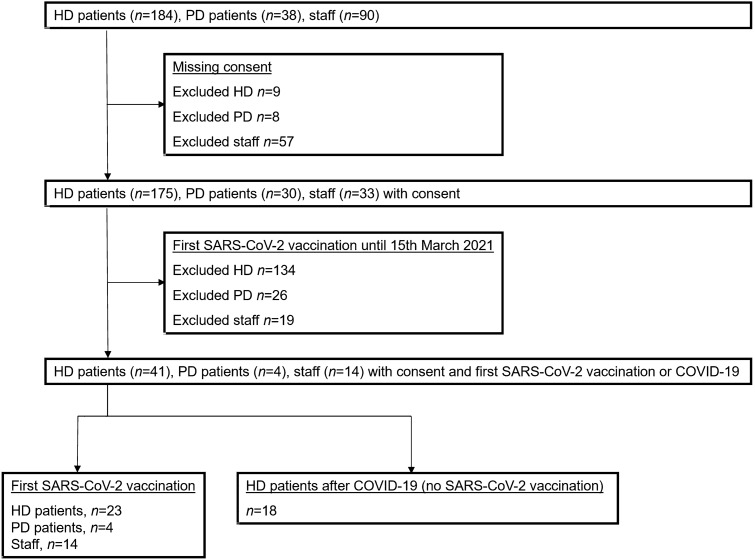
Patient flow through the study. *HD,* hemodialysis. *PD*, peritoneal dialysis

The 59 participants enrolled in the study included 41 individuals receiving regular hemodialysis, four on peritoneal dialysis, and 14 staff (Fig. 1). Of the hemodialysis patients, 23 received a first dose of the SARS-CoV-2 vaccine (Comirnaty N = 11, Vaxzevria N = 12) and had no recent COVID-19 infection, whereas 18 had a history of COVID-19 but did not receive a SARS-CoV-2 vaccine. Table 1 summarizes the demographic information of vaccinated hemodialysis patients regarding the SARS-CoV-2 vaccine, antibody levels and neutralization capacity before vaccination, previous vaccinations/immunosuppression, dialysis characteristics, comorbidities, medication and routine laboratory values. In other words, a typical cohort with considerable comorbidity and evidence of immunosuppression but with no significant antibody levels against SARS-CoV-2 prior to vaccination.

**Table 1:** Baseline characteristics

	Hemodialysis patients after 1st mRNA- or vector-based SARS-CoV-2 vaccination N = 23
Age	64 (61–83)
Female	8 (34.8%)
Vintage (months)	26 (13–50)
Body mass index (kg/m^2^)	27.5 (25.3–30.8)
Nursing home	2 (8.7%)
Disability	4 (17.4%)
Tobacco use	1 (4.4%)
Alcohol abuse disorder	3 (13.0%)
Drug abuse disorder	0 (0%)
SARS-CoV-2 vaccination-related information	
1st SARS-CoV-2 vaccination (comirnaty/vaxzevria)	11 (47.8%)/12 (52.2%)
Interval between 1st SARS-CoV-2 vaccination and sampling, days	14 (13–16)
Hospitalization within 14 days after 1st SARS-CoV-2 vaccination	0 (0%)
Antibody levels and neutralization capacity before vaccination^a^	
IgG spike, AU/mL	0.0 (0.0–0.8)
IgM spike, index	0.03 (0.02–0.04)
IgG nucleocapsid, index	0.05 (0.02–0.08)
ACE2 receptor binding inhibition, %	4.4 (3.1–5.9)
Previous vaccinations or immunosuppression	
Other vaccines within 14 days	0 (0%)
Time from previous vaccination to SARS-CoV-2 vaccination, months	4.5 (2.8–5.0)
Potential immunosuppression	3 (13.0%)
History of kidney transplantation	5 (21.7%)
Immunodeficiency disorder (other than kidney transplantation)	3 (13.0%)
Dialysis-related information	
Charlson comorbidity index	4.0 (3.0–5.5)
Diabetic nephropathy	3 (13.0%)
Hypertensive kidney disease	12 (52.2%)
Glomerulonephritis	2 (8.7%)
Autosomal dominant polycystic kidney disease	2 (8.7%)
Other/unknown primary kidney disease	4 (17.4%)
Kt/V	1.8 (1.5–2.0)
Fistula	17 (73.9%)
Graft	3 (13.0%)
Central venous catheter	3 (13.0%)
Comorbidities	
Number of comorbidities	16 (13–21)
Transplantation candidate	13 (56.5%)
Obesity (body mass index > 30)	2 (8.7%)
Diabetes mellitus	6 (26.1%)
Hypertension	22 (95.7%)
Ischemic heart disease	8 (34.8%)
Congestive heart failure	8 (34.8%)
Chronic obstructive disease	4 (17.4%)
Stroke/cerebrovascular disorder	3 (13.0%)
Peripheral vascular disease	2 (8.7%)
History of malignancy	7 (30.4%)
Thyroid disorder	8 (34.8%)
Medications	
Erythropoiesis stimulating agents dose (unit per week)	4,000 (550–10,000)
Iron dose (mg/week)	40 (10–50)
Angiotensin blockers	12 (52.2%)
ACE inhibitors	4 (17.4%)
Betablockers	19 (82.6%)
Calcium antagonists	12 (52.2%)
Diuretics	14 (60.9%)
Phosphate binders	13 (56.5%)
Insulin	4 (17.4%)
Vitamin D	17 (73.9%)
Active vitamin D	14 (60.9%)
Laboratory values	
Albumin g/L	37 (33–41)
Hypoalbuminemia (albumin < 3.5 g/L)	10 (43.5%)
Hemoglobin (g/dL)	11.1 (10.7–11.4)
Transferrin saturation (%)	25.0 (19.7–29.0)
Ferritin (mg/dL)	422 (271–484)
White blood cell count	5.9 (5.4–7.2)
C-reactive protein, ml/L	4 (2–10)

SARS-CoV-2, severe acute respiratory syndrome coronavirus 2

^a^Missing values N = 14

Peritoneal dialysis patients were aged 60 (52–79) years (all females, one Comirnaty/three Vaxzevria). All staff received Vaxzevria (age 54, [35–56] years, 13 females).

Time from first vaccination to sampling was 14 (13–16) days for hemodialysis patients, 17.5 (13.3–21.0) days for peritoneal dialysis patients and 14 (14–17.5) days for staff.

Time from diagnosis of prior COVID-19 infection to sampling was 6 months (2.5–12.0).

### Effect of 1st vaccination in hemodialysis patients versus peritoneal dialysis patients and staff

None of the vaccinated hemodialysis or peritoneal patients or staff was positive for the IgG Nucleocapsid-protein, indicating immunity did not result from a recent COVID-19 infection. Vaccination responder rates were 17.4% (4/23) in hemodialysis patients, 100% (4/4) in peritoneal dialysis patients and 57.1% (8/14) in staff (HD vs. PD: p = 0.004, HD vs. staff: p = 0.027).

**Fig. 2:**
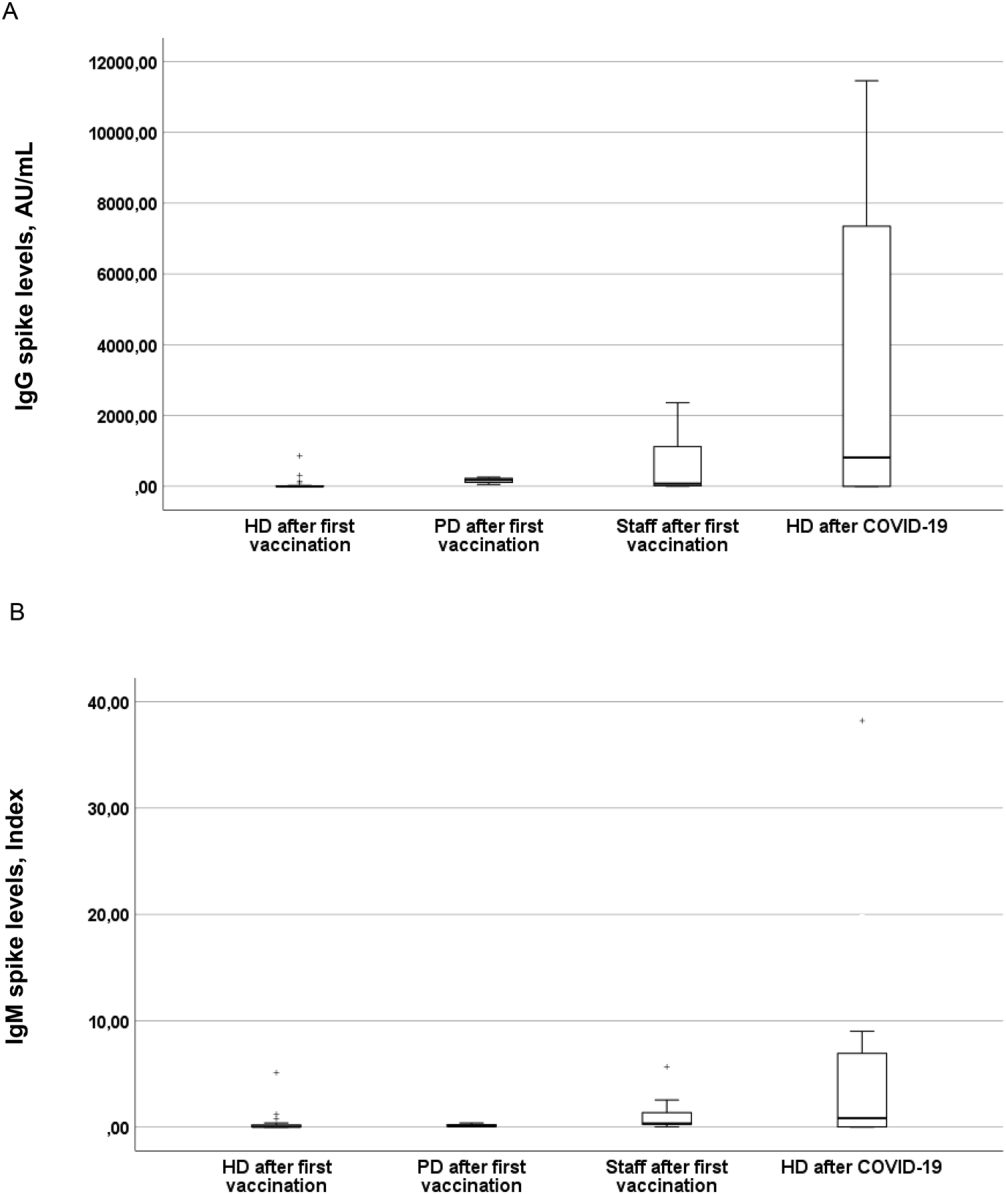
SARS-CoV-2 IgG (**A**) and IgM (**B**) spike levels in HD and PD patients and staff 2 weeks after first vaccination and in HD patients after COVID-19 infection. **A** Median (1;3 quartile). HD patients 2 weeks after first vaccination (N = 23): 1.6 (0–14.5) AU/mL. PD patients 2 weeks after first vaccination (N = 4): 180.7 (82.5–241.9) AU/mL. Staff 2 weeks after first vaccination (N = 14): 73.1 (16.1–1324.5) AU/mL. HD patients after COVID-19 (N = 18): 818.4 (1.6–7806.1) AU/mL. **B** Median (1;3 quartile). HD patients 2 weeks after first vaccination (N = 23): 0.04 (0.03–0.21) Index. PD patients 2 weeks after first vaccination (N = 4): 0.08 (0.07–0.32) Index. Staff 2 weeks after first vaccination (N = 14): 0.34 (0.22–1.67) Index. HD patients after COVID-19 (N = 18): 0.86 (0.03–7.46) Index. Missing values IgG and IgM spike: N = 0. *HD,* hemodialysis. *PD*, peritoneal dialysis

Figure 2 shows lower IgG and IgM spike levels in first vaccinated hemodialysis patients compared to the levels in peritoneal dialysis patients and staff (IgG spike: HD 1.6 [0–14.5] vs. PD 180.7 [82.5–241.9] AU/mL, p = 0.011; HD versus staff 73.1 [16.1–1324.5] AU/mL, p < 0.001, missing values N = 0). ACE2-receptor-binding-inhibition capacity was low in vaccinated hemodialysis (5.0% [3.1–10.4]) and peritoneal dialysis patients (12.9% [9.6–19.8]) and in staff (10.5% [6.0–40.9]), as well as in hemodialysis patients responding to vaccination (11.5% [5.0–27.3]).

### Effect of mRNA- versus vector-based SARS-CoV-2 vaccine in hemodialysis patients

**Fig. 3:**
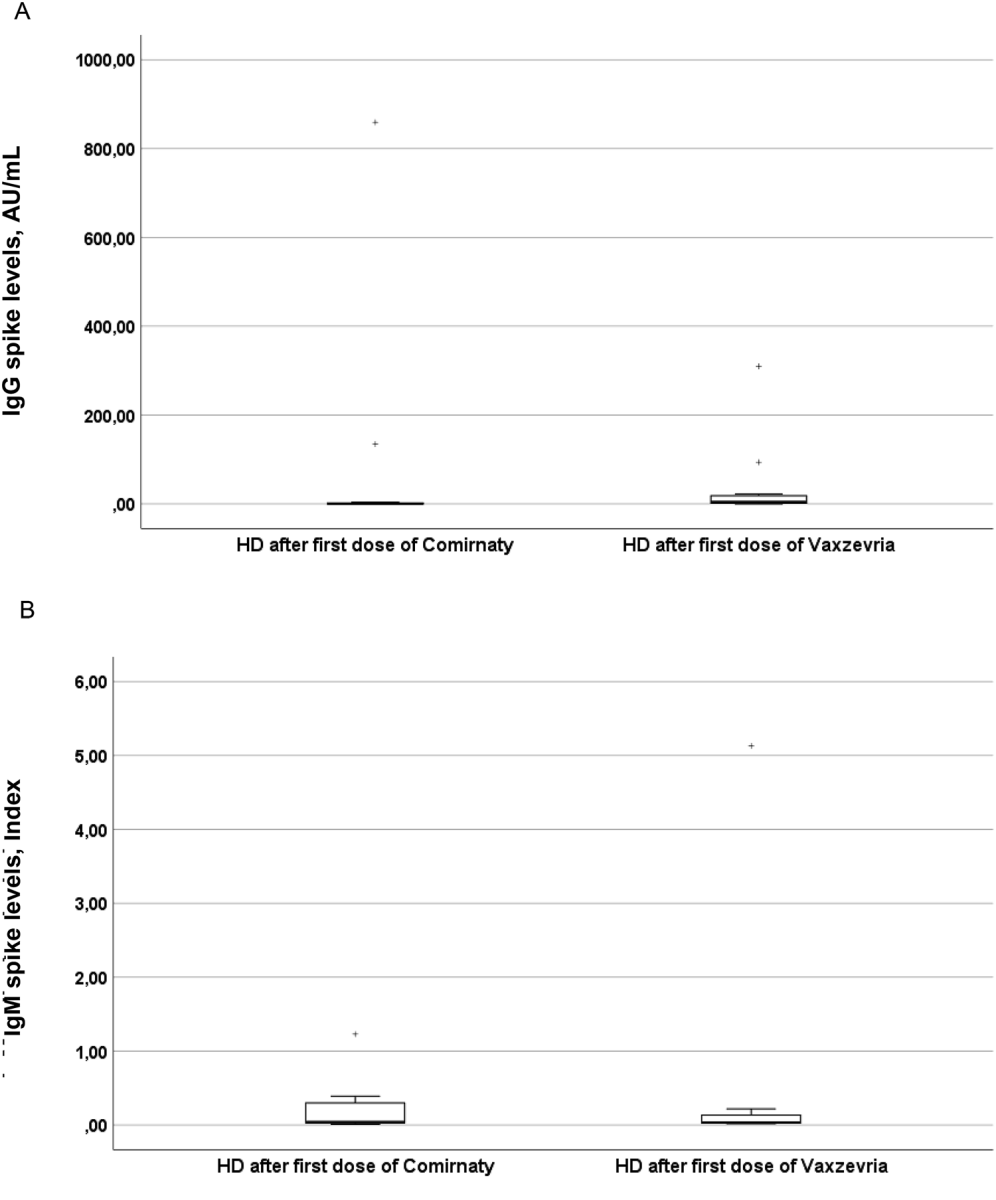
Response of HD patients to different types of vaccinations—SARS-CoV-2 IgG (**A**) and IgM (**B**) spike levels 2 weeks after first vaccination. **A** IgG Median (1;3 quartile): Comirnaty (N = 11): 0.0 (0.0–3.8) AU/mL. Vaxzevria (N = 12): 4.3 (1.6–20.1) AU/mL. p = 0.079. **B** IgM Median (1;3 quartile): Comirnaty (N = 11): 0.04 (0.03–0.39) Index. Vaxzevria (N = 12): 0.04 (0.02–0.17) Index. p = 0.786. Missing values IgG and IgM spike: N = 0, *HD,* hemodialysis

Time from vaccination to sampling was 13.0 (13.0–16.0) days for Comirnaty (N = 11) and 14.5 (14.0–16.8) days for Vaxzevria (N = 12), with two responders to each vaccination. Type of vaccine did not appear to influence the antibody levels of hemodialysis patients (Fig. 3A,B; missing values N = 0). ACE2-receptor-binding-inhibition was 3.8% (1.1–11.3) in hemodialysis patients receiving Comirnaty and 7.1% (3.8–14.9) in those receiving Vaxzevria.

### First vaccinated hemodialysis patients versus hemodialysis patients with previous COVID-19 infection

Of the 18 hemodialysis patients with prior COVID-19 infection, 15 were positive for the IgG Nucleocapsid-protein. IgM spike levels were 0.86 (0.03–7.46) and Nucleocapsid-protein Index levels were 3.0 (1.4–6.0).

Of the hemodialysis patients with prior COVID-19 infection, those with IgG spike-positivity (13/18, 72.2%) had higher IgG spike levels and ACE2-receptor-binding-inhibition capacity compared with that of first dose vaccination-responding hemodialysis patients (N = 4/23): IgG spike: 3,794.6 (793.4–9357.9) vs. 222.0 (104.1–721.9) AU/mL, p = 0.015; ACE2-receptor-binding-inhibition capacity: 74.8% (44.9–98.1) vs. 11.5% (5.0–27.3), p = 0.002.

### SARS-CoV-2 IgG spike levels and ACE2-receptor-binding-inhibition capacity

There was high correlation between reported IgG spike levels and ACE2-receptor-binding-inhibition capacity (Spearman correlation coefficient r = 0.89, p < 0.001).

## Discussion

Two weeks after the first SARS-CoV-2 vaccine dose, we demonstrated a low responder rate and minimal neutralizing antibody levels in hemodialysis patients regardless of the type of vaccine. Following prior COVID-19 infection in hemodialysis patients, antibody-related immunity was more pronounced than that of responding first vaccinated hemodialysis patients. There was a strong correlation between SARS-CoV-2 IgG spike levels and ACE2-receptor-binding-inhibition capacity.

Notably, COVID-19 occurring in the normal population shortly after first vaccination has been described. A recent study reported that, as vaccination programs start to roll out, social distancing decreases due to the anticipated efficacy of SARS-CoV-2 vaccinations [6]. Recently, reduced antibody response after the first dose of mRNA-based COVID-19 vaccine in hemodialysis patients was briefly reported [7]. However, the effect of the first dose of a vector-based vaccine in hemodialysis patients remains unknown and peritoneal dialysis patients have not yet been investigated in this regard.

The findings of the present study are novel regarding the severely impaired quantitative and qualitative antibody-related response in hemodialysis patients 2 weeks after the first dose of both mRNA- and vector-based vaccines. To prevent new cases of COVID-19 between the first and second vaccination, our study findings suggest that SARS-CoV-2 protective measures should at least be sustained in dialysis patients and staff until the full effect of the second vaccination dose is achieved. Study results also imply that hemodialysis patients should not be considered for delayed second dose of vaccination. Our study might point toward more rapid vaccination response in peritoneal dialysis patients. The high antibody levels in patients with prior COVID-19 infection confirms previous findings in patients on maintenance dialysis who recovered from COVID-19 [8]. Whether this persistent immunity may predispose dialysis patients to a similar triggering effect of a single vaccine dose as described for other populations, remains to be studied [9]. The demonstrated strong association between IgG spike levels and ACE2-receptor-binding-inhibition elicited by a single vaccine dose in dialysis patients was previously shown in patients with COVID-19 [10] and may confirm assay usability in dialysis patients.

### Strengths and limitations

The generalizability of our study results is limited by the small patient number. Cell-related immunity was not measured. However, we report the findings of a prospective multicenter pilot study including results of a neutralizing antibody assay and have taken advantage of the use of different types of vaccinations in our hemodialysis patients. Furthermore, healthy staff after first vaccination dose and hemodialysis patients after COVID-19 infection were reported as control groups.

In conclusion, most hemodialysis patients and a considerable proportion of healthy staff exhibited an insufficient antibody-related response 2 weeks after a first mRNA- or vector-based SARS-CoV-2 vaccination, indicating the need to continue with protective measures. Patients with prior COVID-19 infection demonstrated a persistence of SARS-CoV-2 spike-protein antibodies. Further studies with serial measurements of spike-protein antibodies are needed to determine whether this antibody persistence may result in a triggering effect of a single vaccine dose, thus potentially saving the need for a second dose in hemodialysis patients with prior COVID-19.

## Data Availability

The datasets generated and/or analyzed during the current study are available from the corresponding author on reasonable request.
